# A Mixed Finite Element Method for Stationary Magneto-Heat Coupling System with Variable Coefficients

**DOI:** 10.3390/e24070912

**Published:** 2022-06-30

**Authors:** Qianqian Ding, Xiaonian Long, Shipeng Mao

**Affiliations:** 1 School of Mathematics, Shandong University, Jinan 250100, China; dingqianqian@lsec.cc.ac.cn; 2 College of Mathematics and Information Science, Henan University of Economics and Law, Zhengzhou 450045, China; 3 NCMIS, LSEC, Institute of Computational Mathematics and Scientific/Enginnering Computing, Academy of Mathematics and Systems Science, Chinese Academy of Sciences, School of Mathematical Science, University of Chinese Academy of Sciences, Beijing 100190, China; maosp@lsec.cc.ac.cn

**Keywords:** magnetohydrodynamics, variable coefficients, mixed element method, uniqueness, partial differential equations, stationary flows, error analysis

## Abstract

In this article, a mixed finite element method for thermally coupled, stationary incompressible MHD problems with physical parameters dependent on temperature in the Lipschitz domain is considered. Due to the variable coefficients of the MHD model, the nonlinearity of the system is increased. A stationary discrete scheme based on the coefficients dependent temperature is proposed, in which the magnetic equation is approximated by Nédélec edge elements, and the thermal and Navier–Stokes equations are approximated by the mixed finite elements. We rigorously establish the optimal error estimates for velocity, pressure, temperature, magnetic induction and Lagrange multiplier with the hypothesis of a low regularity for the exact solution. Finally, a numerical experiment is provided to illustrate the performance and convergence rates of our numerical scheme.

## 1. Introduction

In this paper, we develop a steady-state magnetohydrodynamics (MHD) system coupling thermal problem with the physical parameters dependent on temperature in $R^{3}$ [1,2,3], as follows:

(1) $$\begin{array}{cc} \; & -\mathrm{div}\;[\nu (\theta )\nabla u]+(u\cdot \nabla )u+\nabla p+\mu B\times \mathrm{curl}\;B-\beta (\theta )\theta =f\;\mathrm{in}\Omega , \end{array}$$

(2) $$\begin{array}{cc} \; & \mathrm{curl}\;[\sigma (\theta )\mathrm{curl}\;B]-\mathrm{curl}\;(u\times B)-\nabla r=g\;\mathrm{in}\Omega , \end{array}$$

(3) $$\begin{array}{cc} \; & -\mathrm{div}\;[\kappa (\theta )\nabla \theta ]+u\cdot \nabla \theta =\psi \;\mathrm{in}\Omega , \end{array}$$

(4) $$\begin{array}{cc} \; & \mathrm{div}\;u=0\;\mathrm{in}\Omega , \end{array}$$

(5) $$\begin{array}{cc} \; & \mathrm{div}\;B=0\;\mathrm{in}\Omega , \end{array}$$

(6) $$\begin{array}{cc} \; & u=0,\;\theta =0,\;B\times n=0,\;r=0\;\mathrm{on}\partial \Omega , \end{array}$$

where n is the outer unit normal of ∂Ω, and Ω is a bounded domain with a Lipschitz boundary ∂Ω; (u, p, B, *r*, θ) denote the velocity field, pressure, magnetic induction, scalar fuction and temperature; (ν, σ, μ, κ, β) denote the kinematic viscosity, electric conductivity, coupling number, thermal conductivity and thermal expansion coefficient; (ψ, f, g) denote a given heat source, a forcing term for magnetic induction and the known applied current with divg=0. In fact, the scalar function *r* is a virtual function. The purpose of adding *r* is related to the constraint divB=0 [4,5].

Many numerical methods for incompressible MHD problems have been widely studied. According to our investigation, a considerable number of scholars in the literature use continuous Lagrange finite elements to approximate velocity and magnetic induction unknowns, see, e.g., [6,7,8,9,10]. However, it is well known that using continuous elements to approximate magnetic induction may lead to an inaccurate approximation when the regularity of the magnetic unknown is lower than $H^{1}\left(\Omega \right)$, cf. [11,12], which may often be encountered in non-convex polyhedral or with a non-$C^{1,1}$ boundary. A novel method to overcome these shortcomings was put forward in [13] by using Nédélec finite elements to approximate the magnetic unknown ***B***. This seems attractive and has been used in [14,15,16,17] and the references therein. In addition, the authors had proposed different formulas to keep the magnetic solutions divergence-free in the numerical schemes in the papers [18,19].

Because of the movement of the fluid with viscosity, viscosity will produce heat. Under the action of the external magnetic field, the incompressible MHD problem is usually coupled with the thermal system through the famous Boussinesq approximation. For example, Meir et al. [20,21] studied the mixed finite element method for the thermally coupled MHD equation by adopting continuous elements to approximate the unknowns of magnetic, fluid and thermal problems, which is pioneering work. Coupling fluid systems or electromagnetic models with coefficients dependent on temperature face the mathematical challenge of solving strongly nonlinear partial differential equations, as studied by some physicists and mathematicians, see, e.g., [22,23,24,25,26,27,28,29] and the references therein. In addition, in many practical applications of the MHD system, the change in temperature will lead to a change in the coefficients in the fluid field and electromagnetic field. For the coupling MHD problem whose coefficients depend on temperature, it is important to study its reliable finite element method.

In this paper, we aim to give a rigorous well-posed analysis of the solution to the continuous problem and error estimates for the MHD system with variable coefficients by the finite element method. The fluid field was approximated by Taylor–Hood-type finite elements, and the thermal system was approximated by Lagrange finite elements. Considering the highly nonlinearity brought by the physical parameters dependent on temperature and the Lorentz terms in the magnetic equation, as well as a possible non-convex domain or a non-$C^{1,1}$ boundary, we choose H(curl)-conforming Nédélec edge elements to approximate the magnetic equation to capture the physical solutions. The optimal error estimates of velocity, pressure, temperature, magnetic induction and Lagrange multiplier are established. As far as we know, there still lacks rigorous analysis in the literature for the error estimate of the stationary MHD thermally coupled model with temperature-dependent coefficients.

The remaining parts of this paper are organized as follows. In Section 2, we introduce some notations and basic finite element estimation used in the discussion and show the uniqueness of the solution to the continuous system. In Section 3, we propose a discrete finite element method for the variable coefficient system consisting of Equations (1)–(6). In Section 4, we give the convergence of all variables under the slightly smooth regularity assumption. In Section 5, we verify the effectiveness of the proposed numerical method through a numerical experiment.

## 2. Notations for the Variable Coefficients Model

Firstly, we introduce some symbols that will be used throughout this article. For all $m\in N^{+}$, 1≤p≤∞, let $W^{m,p}\left(\Omega \right)$ denote the standard Sobolev space, and when p=2, it can be written as $H^{m}\left(\Omega \right)$. The notation (·,·) is expressed as an inner product, namely $\left(\phi ,\psi \right)=\int_{\Omega }\phi \psi \;dx$, and the norm in $L^{2}\left(\Omega \right)$ defined by ${∥\cdot ∥}_{0}$. Vector-valued quantities will be denoted in boldface notations, such as $u=(u_{1},u_{2},u_{3})$ and $L^{2}\left(\Omega \right)={\left(L^{2}\left(\Omega \right)\right)}^{3}$. We use *C* and *c* to denote generic positive constants independent of the mesh size *h*, and it may adopt different values in different places.

To simplify, we define the following Sobolev spaces

$$\begin{array}{cc} \; & X=H_{0}^{1}\left(\Omega \right),\;Y=H_{0}^{1}\left(\Omega \right),\;Q=\left\{q\in L^{2}\left(\Omega \right),\;\int_{\Omega }q\left(x\right)\;dx=0\right\},\\ \; & W=\left\{C\in L^{2}\left(\Omega \right),\mathrm{curl}\;C\in L^{2}\left(\Omega \right)\right\},\;W_{0}=\left\{C\in W,\;\;C\times n{|}_{\partial \Omega }=0\right\}. \end{array}$$

The norm of the following types still need to be defined:

$${∥C∥}_{W}={∥C∥}_{H(\mathrm{curl};\Omega )}={\left({∥C∥}_{0}^{2}+{∥\mathrm{curl}\;C∥}_{0}^{2}\right)}^{1/2}\;\forall \;C\in W.$$

We then set

$$H\left(\mathrm{div};\Omega \right)=\{b\in L^{2}\left(\Omega \right),\mathrm{div}\;b\in L^{2}\left(\Omega \right)\},$$

$$H\left({\mathrm{div}}^{0};\Omega \right)=\left\{b\in H\left(\mathrm{div};\Omega \right),\mathrm{div}\;b=0\right\}$$

and $H\left(\Omega \right)=W_{0}\cap H\left(\mathrm{div};\Omega \right)$, which is equipped with the following norm:

$${∥v∥}_{H(\Omega )}={\left({∥\mathrm{curl}\;v∥}_{0}^{2}+{∥\mathrm{div}\;v∥}_{0}^{2}\right)}^{1/2}\;\forall \;v\in H\left(\Omega \right).$$

To facilitate our analysis, the following embedding results (see, e.g., Proposition 3.7 of [30] or [31]) are introduced here, which are also valid for the Lipschitz polyhedron domain.

**Lemma** **1.** *There exists a parameter $\delta_{1}=\delta_{1}\left(\Omega \right)>0$ such that the embedding $H\left(\Omega \right)↪L^{3+\delta_{1}}\left(\Omega \right)$ is compact.*

In order to better demonstrate the stability of energy, the following trilinear terms are denoted

$$\begin{array}{cc} O_{1}\left(w,u,v\right)= & \frac{1}{2}\left\{\int_{\Omega }\left[\left(w\cdot \nabla \right)u\right]\;v\;dx-\int_{\Omega }\left[\left(w\cdot \nabla \right)v\right]\;u\;dx\right\},\\ O_{3}\left(u,\theta ,\varphi \right)= & \frac{1}{2}\left\{\int_{\Omega }\left(u\cdot \nabla \theta \right)\varphi \;dx-\int_{\Omega }\left(u\cdot \nabla \varphi \right)\theta \;dx\right\}, \end{array}$$

for any (w,u,v)∈X×X×X and (θ,φ)∈(Y×Y).

Next, the definition of the weak solution to the magneto-heat coupling system with variable coefficients (Equations (1)–(6)) is given.

**Definition** **1.** *Provided that $f\in H^{-1,2}\left(\Omega \right)$, $\psi \in H^{-1,2}\left(\Omega \right)$ and $g\in W^{\prime }$, where $W^{\prime }$ is the dual space of W. We say $\left(u,p,\theta ,B,r\right)\in X\cap H\left({\mathrm{div}}^{0};\Omega \right)\times Q\times Y\times W_{0}\cap H\left({\mathrm{div}}^{0};\Omega \right)\times Y$ is the weak solution of Equations (1)–(6), if there holds*

(7) $$\begin{array}{cc} \; & A_{1}\left(\nu \left(\theta \right),u,v\right)+O_{1}\left(u,u,v\right)+\mu \;O_{2}\left(B,B,v\right)+b\left(v,p\right)-\left(\beta \left(\theta \right)\theta ,v\right)=\left(f,v\right), \end{array}$$

(8) $$\begin{array}{cc} \; & A_{2}\left(\sigma \left(\theta \right),B,C\right)-O_{2}\left(B,C,u\right)+a\left(C,r\right)=\left(g,C\right), \end{array}$$

(9) $$\begin{array}{cc} \; & A_{3}\left(\kappa \left(\theta \right),\theta ,\varphi \right)+O_{3}\left(u,\theta ,\varphi \right)=\left(\psi ,\varphi \right), \end{array}$$

*for any $\left(v,\varphi ,C\right)\in \left(X\times Y\times W_{0}\right)$, where*

$$\begin{array}{cc} \; & A_{1}\left(\nu \left(\theta \right),u,v\right)=\int_{\Omega }\nu \left(\theta \right)\nabla u:\nabla v\;dx,\;b\left(v,q\right)=-\int_{\Omega }q\;\mathrm{div}\;v\;dx,\\ \; & A_{2}\left(\sigma \left(\theta \right),B,C\right)=\int_{\Omega }\sigma \left(\theta \right)\mathrm{curl}\;B\cdot \mathrm{curl}\;C\;dx,\;a\left(C,r\right)=-\int_{\Omega }\nabla r\cdot C\;dx,\\ \; & O_{2}\left(B,C,u\right)=\int_{\Omega }B\times \mathrm{curl}\;C\cdot u\;dx,\;A_{3}\left(\kappa \left(\theta \right),\theta ,\varphi \right)=\int_{\Omega }\kappa \left(\theta \right)\nabla \theta \cdot \nabla \varphi \;dx. \end{array}$$

**Remark** **1.** *Owing to $\nabla \phi \in W_{0}$ for any $\phi \in H_{0}^{1}\left(\Omega \right)$, by selecting C=∇ϕ in Equation (8), it can be derived −(∇r,∇ϕ)=0. Therefore, the Lagrange multiplier r=0 in the sense of a weak formulation. In this case, the corresponding orthogonal decomposition is $L^{2}\left(\Omega \right)=H\left({\mathrm{div}}^{0}\;;\Omega \right)⊕\nabla H_{0}^{1}\left(\Omega \right)$, see [32,33].*

**Remark** **2.** *The existence of a solution to the system in Equations (7)–(9) can be referred to the work of [34] (Section 4), albeit under additional smoothness assumptions on the domain. Here, we restrict ourselves to proving the following (more straightforward) uniqueness result.*

### Uniqueness of Continuous Problems

Throughout the paper, we set that σ(θ), κ(θ), β(θ) and ν(θ) are Lipschitz continuous and satisfy $0<\sigma_{0}\leq \sigma \left(\theta \right)\leq \sigma_{1}$, $0<\kappa_{0}\leq \kappa \left(\theta \right)\leq \kappa_{1}$, $0<\beta_{0}\leq \beta \left(\theta \right)\leq \beta_{1}$ and $0<\nu_{0}\leq \nu \left(\theta \right)\leq \nu_{1}$.

Before proving the uniqueness of continuous problems, we first introduce some basic knowledge.

**Lemma** **2.** *For any u, B and θ that satisfy Equations (7)–(9), the following estimates hold*

$$\begin{array}{cc} A_{1}\left(\nu \left(\theta \right),u,v\right) & \leq \nu_{1}{∥\nabla u∥}_{0}{∥\nabla v∥}_{0},\\ A_{1}\left(\nu \left(\theta \right),u,u\right) & \geq \nu_{0}{∥\nabla u∥}_{0}^{2},\\ A_{2}\left(\sigma \left(\theta \right),B,C\right) & \leq \sigma_{1}{∥\mathrm{curl}\;B∥}_{0}{∥\mathrm{curl}\;C∥}_{0},\\ A_{2}\left(\sigma \left(\theta \right),B,B\right) & \geq \sigma_{0}{∥\mathrm{curl}\;B∥}_{0}^{2},\\ O_{1}\left(u,u,v\right) & \leq C_{*}{∥\nabla u∥}_{0}{∥\nabla u∥}_{0}{∥\nabla v∥}_{0},\\ O_{3}\left(u,\theta ,\varphi \right) & \leq C_{*}{∥\nabla u∥}_{0}{∥\nabla \theta ∥}_{0}{∥\nabla \varphi ∥}_{0},\\ A_{3}\left(\kappa \left(\theta \right),\theta ,\varphi \right) & \leq \kappa_{1}{∥\nabla \theta ∥}_{0}{∥\nabla \varphi ∥}_{0},\\ A_{3}\left(\kappa \left(\theta \right),\theta ,\theta \right) & \geq \kappa_{0}{∥\nabla \theta ∥}_{0}^{2}. \end{array}$$

*Further, the skew-symmetry $O_{1}\left(u,u,u\right)=0$ and $O_{3}\left(u,\theta ,\theta \right)=0$ hold.*

For the convenience of the subsequent analysis, setting

$${∥l∥}_{*}={\left[2\nu_{0}^{-1}{∥f∥}_{-1,2}^{2}+2\nu_{0}^{-1}\beta_{1}^{2}\kappa_{0}^{-2}{∥\psi ∥}_{-1,2}^{2}+\mu \sigma_{0}^{-1}{∥g∥}_{W^{\prime }}^{2}+\kappa_{0}^{-1}{∥\psi ∥}_{-1,2}^{2}\right]}^{1/2}.$$

The norms are defined as:

$$\begin{array}{cc} |\;|\;|(u,B)|\;|\;|= & {\left({∥\nabla u∥}_{0}^{2}+{∥\mathrm{curl}\;B∥}_{0}^{2}\right)}^{\frac{1}{2}},\\ |\;|\;|(u,B,\theta )|\;|\;|= & {\left({∥\nabla u∥}_{0}^{2}+{∥\mathrm{curl}\;B∥}_{0}^{2}+{∥\nabla \theta ∥}_{0}^{2}\right)}^{\frac{1}{2}},\\ |\;|\;|(p,r)|\;|\;|= & {\left({∥p∥}_{0}^{2}+{∥\nabla r∥}_{0}^{2}\right)}^{\frac{1}{2}}. \end{array}$$

It is well known that (cf. [31,35]) both $H_{0}^{1}\left(\Omega \right)\times Q$ and $W\times H_{0}^{1}\left(\Omega \right)$ satisfy the corresponding inf-sup conditions, namely,

(10) $$\underset{0\neq q\in Q}{\inf}\underset{0\neq v\in H_{0}^{1}\left(\Omega \right)}{\sup}\frac{\left(\mathrm{div}\;v,q\right)}{{∥v∥}_{1,2}{∥q∥}_{0}}\geq C,$$

and

(11) $$\underset{0\neq j\in H_{0}^{1}\left(\Omega \right)}{\inf}\underset{0\neq C\in W}{\sup}\frac{\left(\nabla j,C\right)}{{∥C∥}_{H(\mathrm{curl};\Omega )}{∥\nabla j∥}_{0}}\geq C.$$

where the generic constant *C* only depends on Ω.

Before we begin the proof, the following assumptions should be given:

- $|A_{1}\left(\nu \left(\theta_{1}\right)-\nu \left(\theta_{2}\right),u,u\right)|\leq C_{lip}∥\nabla \left(\theta_{1}-\theta_{2}\right){∥}_{0}{∥\nabla u∥}_{0,3}{∥\nabla u∥}_{0}$,
- $|A_{2}\left(\sigma \left(\theta_{1}\right)-\sigma \left(\theta_{2}\right),B,B\right)|\leq C_{lip}∥\nabla \left(\theta_{1}-\theta_{2}\right){∥}_{0}{∥\mathrm{curl}\;B∥}_{0,3}{∥\mathrm{curl}\;B∥}_{0}$,
- $|A_{3}\left(\kappa \left(\theta_{1}\right)-\kappa \left(\theta_{2}\right),\theta ,\theta \right)|\leq C_{lip}∥\nabla \left(\theta_{1}-\theta_{2}\right){∥}_{0}{∥\nabla \theta ∥}_{0,3}{∥\nabla \theta ∥}_{0}$,

where $C_{lip}$ is the Lipschitz constant.

Now, we are going to prove that the solution of the continuous problem is unique.

**Theorem** **1.** *Suppose that there exists a sufficiently small constant γ>0 such that $\max\{∥\nabla u{∥}_{0,3},∥c\;B{∥}_{0,3},∥\nabla \theta {∥}_{0,3}\}\leq \gamma$ (A precise condition on γ can be found in Equation (19)), then the solution of Equations (7)–(9) is well-posed. In addition,*

(12) $$\left|\;|\;|\left(u,B,\theta \right)|\;|\;\right|\leq \frac{{∥l∥}_{*}}{\min\{\nu_{0},\mu \sigma_{0},\kappa_{0}\}}.$$

**Proof.**  Taking φ=2θ in Equation (9), we can deduce that

$$\begin{array}{cc} \; & {2\kappa \left(\theta \right)∥\nabla \theta ∥}_{0}^{2}=2\left(\psi ,\theta \right)\leq {\kappa \left(\theta \right)∥\nabla \theta ∥}_{0}^{2}+\kappa_{0}^{-1}{∥\psi ∥}_{-1,2}^{2}, \end{array}$$

this implies

(13) $$\begin{array}{cc} \; & {∥\nabla \theta ∥}_{0}^{2}\leq \kappa_{0}^{-2}{∥\psi ∥}_{-1,2}^{2}. \end{array}$$

Setting v=2u,C=2μB in Equations (7) and (8), we have

$$\begin{array}{cc} \; & 2A_{1}\left(\nu \left(\theta \right),u,u\right)+2\mu \left(\sigma \left(\theta \right)\mathrm{curl}\;B,\mathrm{curl}\;B\right)=2\left(f,u\right)+2\left(\beta \left(\theta \right)\theta ,u\right)+2\mu \left(g,B\right), \end{array}$$

combining with Equation (13), there holds

$$\begin{array}{cc} \; & \nu_{0}{∥\nabla u∥}_{0}^{2}+\mu \sigma_{0}{∥\mathrm{curl}\;B∥}_{0}^{2}\leq 2\nu_{0}^{-1}{∥f∥}_{-1,2}^{2}+2\nu_{0}^{-1}\beta_{1}^{2}\kappa_{0}^{-2}{∥\psi ∥}_{-1,2}^{2}+\mu \sigma_{0}^{-1}{∥g∥}_{W^{\prime }}^{2}, \end{array}$$

which implies that

(14) $$\min\left\{\nu_{0},\mu \sigma_{0}\right\}\left|\;|\;\right|\left(u,B\right)\left|\;|\;\right|\leq {∥l∥}_{*}.$$

Then, the following estimation is valid

(15) $$\left|\;|\;|\left(u,B,\theta \right)|\;|\;\right|\leq \frac{{∥l∥}_{*}}{\min\{\nu_{0},\mu \sigma_{0},\kappa_{0}\}}.$$

Now, we start to demonstrate the uniqueness of the solution for the problem in Equations (7)–(9). Suppose that $\left(u_{1},p_{1},B_{1},r_{1},\theta_{1}\right)$ and $\left(u_{2},p_{2},B_{2},r_{2},\theta_{2}\right)$ are two arbitrary solution of Equations (7)–(9), for any $\left(v,C,\varphi \right)\in X\times W_{0}\times Y$ such that

(16) $$\begin{array}{c} A_{1}\left(\nu \left(\theta_{1}\right),u_{1},v\right)+O_{1}\left(u_{1},u_{1},v\right)+\mu \left(B_{1}\times \mathrm{curl}\;B_{1},v\right)\\ -\mu \left(u_{1}\times B_{1},\mathrm{curl}\;C\right)+\mu \left(\sigma \left(\theta_{1}\right)\mathrm{curl}\;B_{1},\mathrm{curl}\;C\right)\\ +A_{3}\left(\kappa \left(\theta_{1}\right),\theta_{1},\varphi \right)+O_{3}\left(u_{1},\theta_{1},\varphi \right)+b\left(v,p_{1}\right)\\ +\mu \;a\left(C,r_{1}\right)=\left(f,v\right)+\left(\beta \left(\theta_{1}\right)\theta_{1},v\right)+\mu \left(g,C\right)+\left(\psi ,\varphi \right) \end{array}$$

and

(17) $$\begin{array}{c} A_{1}\left(\nu \left(\theta_{2}\right),u_{2},v\right)+O_{1}\left(u_{2},u_{2},v\right)+\mu \left(B_{2}\times \mathrm{curl}\;B_{2},v\right)\\ -\mu \left(u_{2}\times B_{2},\mathrm{curl}\;C\right)+\mu \left(\sigma \left(\theta_{2}\right)\mathrm{curl}\;B_{2},\mathrm{curl}\;C\right)\\ +A_{3}\left(\kappa \left(\theta_{2}\right),\theta_{2},\varphi \right)+O_{3}\left(u_{2},\theta_{2},\varphi \right)+b\left(v,p_{2}\right)\\ +\mu \;a\left(C,r_{2}\right)=\left(f,v\right)+\left(\beta \left(\theta_{2}\right)\theta_{2},v\right)+\mu \left(g,C\right)+\left(\psi ,\varphi \right). \end{array}$$

By subtracting Equation (17) from Equation (16), we obtain

(18) $$\begin{array}{c} A_{1}\left(\nu \left(\theta_{1}\right)-\nu \left(\theta_{2}\right),u_{1},v\right)+A_{1}\left(\nu \left(\theta_{2}\right),u_{1}-u_{2},v\right)\\ +O_{1}\left(u_{2},u_{1}-u_{2},v\right)+O_{1}\left(u_{1}-u_{2},u_{1},v\right)\\ +\mu \left(B_{2}\times \mathrm{curl}\;\left[B_{1}-B_{2}\right],v\right)+\mu \left(\left[B_{1}-B_{2}\right]\times \mathrm{curl}\;B_{1},v\right)\\ -\mu \left(u_{1}\times \left[B_{1}-B_{2}\right],\mathrm{curl}\;C\right)-\mu \left(\left[u_{1}-u_{2}\right]\times B_{2},\mathrm{curl}\;C\right)\\ +\mu \left(\left[\sigma \left(\theta_{1}\right)-\sigma \left(\theta_{2}\right)\right]\mathrm{curl}\;B_{1},\mathrm{curl}\;C\right)+\mu \left(\sigma \left(\theta_{2}\right)\mathrm{curl}\;\left[B_{1}-B_{2}\right],\mathrm{curl}\;C\right)\\ +A_{3}\left(\kappa \left(\theta_{1}\right)-\kappa \left(\theta_{2}\right),\theta_{1},\varphi \right)+A_{3}\left(\kappa \left(\theta_{2}\right),\theta_{1}-\theta_{2},\varphi \right)\\ +O_{3}\left(u_{2},\theta_{1}-\theta_{2},\varphi \right)+O_{3}\left(u_{1}-u_{2},\theta_{1},\varphi \right)+b\left(v,p_{1}-p_{2}\right)\\ +\mu \;a\left(C,r_{1}-r_{2}\right)=\left(\left[\beta \left(\theta_{1}\right)-\beta \left(\theta_{2}\right)\right]\theta_{1},v\right)+\left(\beta \left(\theta_{2}\right)\left[\theta_{1}-\theta_{2}\right],v\right). \end{array}$$

Taking $\left(v,C\right)=\left(u_{1}-u_{2},B_{1}-B_{2}\right)\in X\cap H\left({\mathrm{div}}^{0};\Omega \right)\times W_{0}\cap H\left({\mathrm{div}}^{0};\Omega \right)$, applying $O_{1}\left(u_{2},u_{1}-u_{2},u_{1}-u_{2}\right)=0$, there hold

$$\begin{array}{c} A_{1}\left(\nu \left(\theta_{1}\right)-\nu \left(\theta_{2}\right),u_{1},u_{1}-u_{2}\right)+A_{1}\left(\nu \left(\theta_{2}\right),u_{1}-u_{2},u_{1}-u_{2}\right)\\ +O_{1}\left(u_{1}-u_{2},u_{1},u_{1}-u_{2}\right)+\mu \left(\left[B_{1}-B_{2}\right]\times \mathrm{curl}\;B_{1},u_{1}-u_{2}\right)\\ -\mu \left(u_{1}\times \left[B_{1}-B_{2}\right],\mathrm{curl}\;\left[B_{1}-B_{2}\right]\right)+\mu \left(\left[\sigma \left(\theta_{1}\right)-\sigma \left(\theta_{2}\right)\right]\mathrm{curl}\;B_{1},\mathrm{curl}\;\left[B_{1}-B_{2}\right]\right)\\ +\mu \left(\sigma \left(\theta_{2}\right)\mathrm{curl}\;\left[B_{1}-B_{2}\right],\mathrm{curl}\;\left[B_{1}-B_{2}\right]\right)+O_{3}\left(u_{1}-u_{2},\theta_{1},\theta_{1}-\theta_{2}\right)\\ +A_{3}\left(\kappa \left(\theta_{1}\right)-\kappa \left(\theta_{2}\right),\theta_{1},\theta_{1}-\theta_{2}\right)+A_{3}\left(\kappa \left(\theta_{2}\right),\theta_{1}-\theta_{2},\theta_{1}-\theta_{2}\right)\\ =\left(\left[\beta \left(\theta_{1}\right)-\beta \left(\theta_{2}\right)\right]\theta_{1},u_{1}-u_{2}\right)+\left(\beta \left(\theta_{2}\right)\left[\theta_{1}-\theta_{2}\right],u_{1}-u_{2}\right), \end{array}$$

which means that

$$\begin{array}{cc} \; & \min\left\{\nu_{0},\mu \sigma_{0},\kappa_{0}\right\}\left|\;|\;\right|\left(u_{1}-u_{2},B_{1}-B_{2},\theta_{1}-\theta_{2}\right){\left|\;|\;\right|}^{2}\\ \leq & -A_{1}\left(\nu \left(\theta_{1}\right)-\nu \left(\theta_{2}\right),u_{1},u_{1}-u_{2}\right)-O_{1}\left(u_{1}-u_{2},u_{1},u_{1}-u_{2}\right)\\ \; & -\mu \left(\left[B_{1}-B_{2}\right]\times \mathrm{curl}\;B_{1},u_{1}-u_{2}\right)+\mu \left(u_{1}\times \left[B_{1}-B_{2}\right],\mathrm{curl}\;\left[B_{1}-B_{2}\right]\right)\\ \; & -\mu \left(\left[\sigma \left(\theta_{1}\right)-\sigma \left(\theta_{2}\right)\right]\mathrm{curl}\;B_{1},\mathrm{curl}\;\left[B_{1}-B_{2}\right]\right)-A_{3}\left(\kappa \left(\theta_{1}\right)-\kappa \left(\theta_{2}\right),\theta_{1},\theta_{1}-\theta_{2}\right)\\ \; & -O_{3}\left(u_{1}-u_{2},\theta_{1},\theta_{1}-\theta_{2}\right)+\left(\left[\beta \left(\theta_{1}\right)-\beta \left(\theta_{2}\right)\right]\theta_{1},u_{1}-u_{2}\right)+\left(\beta \left(\theta_{2}\right)\left[\theta_{1}-\theta_{2}\right],u_{1}-u_{2}\right)\\ \leq & \max\left\{1,\mu \right\}|\;|\;|\left(u_{1}-u_{2},B_{1}-B_{2},\theta_{1}-\theta_{2}\right){\left|\;|\;\right|}^{2}(\frac{{∥l∥}_{*}}{\min\{\nu_{0},\mu \sigma_{0},\kappa_{0}\}}\\ \; & +C_{lip}(∥\nabla u_{1}{∥}_{0,3}+∥\mathrm{curl}\;B_{1}{∥}_{0,3}+∥\nabla \theta_{1}{∥}_{0,3})). \end{array}$$

Thus, if γ satisfies

(19) $$\begin{array}{cc} \; & 3C_{lip}\gamma <\frac{\min\{\nu_{0},\mu \sigma_{0},\kappa_{0}\}}{\max\{1,\mu \}}-\frac{{∥l∥}_{*}}{\min\{\nu_{0},\mu \sigma_{0},\kappa_{0}\}}, \end{array}$$

where $\max\{∥\nabla u_{1}{∥}_{0,3},∥\mathrm{curl}\;B_{1}{∥}_{0,3},∥\nabla \theta_{1}{∥}_{0,3}\}\leq \gamma$, we can deduce $\left|\;|\;\right|\left(u_{1}-u_{2},B_{1}-B_{2},\theta_{1}-\theta_{2}\right){\left|\;|\;\right|}^{2}\leq 0$, and it is easy to check $\left(u_{1},B_{1},\theta_{1}\right)=\left(u_{2},B_{2},\theta_{2}\right)$.

Putting $\left(u_{1},B_{1},\theta_{1}\right)=\left(u_{2},B_{2},\theta_{2}\right)$ in Equation (18), we have

$$\begin{array}{c} b\left(v,p_{1}-p_{2}\right)+\mu \;a\left(C,r_{1}-r_{2}\right)=0, \end{array}$$

∀$\left(v,C\right)\in X\times W_{0}$. Combining with Conditions (10) and (11), we can arrive at $\left(p_{1},r_{1}\right)=\left(p_{2},r_{2}\right)$. We prove that the system in Equations (7)–(9) has a unique solution. This yields the conclusion. □

## 3. Finite Element Analysis for Magneto-Heat Coupling System

In this section, we introduce a mixed finite element approximation of the MHD system coupled thermal problem in Equations (7)–(9). The approximation is based on the Nédélec first family of elements for the discretization of the magnetic induction.

Throughout, the domain Ω is partitioned into a finite number of open non-overlapping subdomains with regular and quasi-uniform meshes $T_{h}$ of mesh-size *h* that partition Ω into tetrahedra *K*. Each tetrahedron *K* is supposed to be the image of a reference tetrahedron $\hat{K}$ under an affine map $F_{K}$. $P_{k}\left(K\right)$ and ${\tilde{P}}_{k}\left(K\right)$ represent the space of polynomials of the total degree at most k≥0 and homogeneous polynomials *k* on *K*, respectively.

Given the generalized Taylor–Hood element $\left(X_{h}^{k},Q_{h}^{k-1}\right)$ with k≥2, where $X_{h}^{k}$ is the *k* order vectorial Lagrange finite element subspace of X, and $Q_{h}^{k-1}$ is the k−1 order scalar Lagrange finite element subspace of *Q*. For k=1, the velocity and pressure pair can be approximated by the well-known stable mini-elements, cf. [31,35,36]. Furthermore, $Y_{h}^{k}$ is the *k* order scalar Lagrange finite element subspace of *Y*, refer to [31,35].

For $D_{k}\left(K\right)$, it denotes the polynomials q in ${\tilde{P}}_{k}\left(K\right)$ that satisfy q(x)·x=0 on *K*. Define the following space

$$\begin{array}{c} N_{k}\left(K\right)=P_{k-1}\left(K\right)⊕D_{k}\left(K\right). \end{array}$$

where 1≤k. Using Nédélec H(curl)-conforming finite element space (see [33,37])

$$W_{h}^{k}=\left\{C\in W_{0},\;C{|}_{K}\in N_{k}\left(K\right)\;\forall \;K\in T_{h}\right\}$$

to approximate the magnetic induction.

Setting $S_{h}^{k}=\left\{C\in H^{1}\left(\Omega \right)\cap L_{0}^{2}\left(\Omega \right),\;C\in P_{k}\left(K\right),\;\forall \;K\in T_{h}\right\}$, and we can define the following weakly divergent space

$$W_{0h}^{k}=\left\{C\in W_{h}^{k},\left(C,\nabla S\right)=0\;\forall \;S\in S_{h}^{k}\right\}.$$

In addition, the following discrete Poincaré–Friedrichs inequality is established

(20) $$∥c_{h}{∥}_{0}\leq C_{p}{∥\mathrm{curl}\;c_{h}∥}_{0}\;\forall \;c_{h}\in W_{0h}^{k},$$

with a constant $C_{p}>0$ independent of the mesh size *h*.

The link between the spaces $W_{0h}^{k}$ and W(Ω) is accomplished by the Hodge mapping Z:H(curl;Ω)→W(Ω) (refer to [12]), where W(Ω)={C∈H(Ω),divC=0inΩ} such that

curlZ(C)=curlC∀C∈W.

Furthermore, there exists l=l(Ω)>0,

(21) $$∥C_{h}-Z\left(C_{h}\right){∥}_{0}\leq ch^{\frac{1}{2}+l}{∥\mathrm{curl}\;C_{h}∥}_{0}\;\forall \;C_{h}\in W_{0h}^{k}.$$

In addition, the discrete kernel space of the divergence operator is given by

$$X_{0h}^{k}=\left\{v_{h}\in X_{h}^{k};\;b\left(v_{h},q_{h}\right)=0\;\forall q_{h}\in Q_{h}^{k-1}\right\}.$$

On a quasi-uniform mesh, there holds (see Theorem 3.2.6 of [38])

(22) $$\begin{array}{ccc} ∥v_{h}{∥}_{m,q} & \leq & C_{inv}h^{ı-m+3(1/q-1/p)}{∥v_{h}∥}_{ı,p}\;\forall \;v_{h}\in X_{h}^{k}, \end{array}$$

where $C_{inv}>0$ is a generic constant independent of the mesh size *h*, *ı* and *m* are two real numbers with 0≤ı≤m≤1, and *p* and *q* are two integers with 1≤p≤q≤∞.

The following discrete inf-sup conditions (see Chapter 2 of [36] or [12]) are founded

(23) $$\underset{0\neq q\in Q_{h}^{k-1}}{\inf}\underset{0\neq v\in X_{h}^{k}}{\sup}\frac{\left(q,\mathrm{div}\;v\right)}{{∥v∥}_{1,2}{∥q∥}_{0}}\geq \beta_{*},$$

(24) $$\underset{0\neq j\in S_{h}^{k}}{\inf}\underset{0\neq C\in W_{h}^{k}}{\sup}\frac{\left(\nabla j,C\right)}{{∥C∥}_{H(\mathrm{curl};\Omega )}{∥j∥}_{1,2}}\geq \beta_{*},$$

where $\beta_{*}$ is a generic positive constant depending on the domain Ω.

For k≥1, our goal is to find $\left\{\left(u_{h},p_{h},\theta_{h},B_{h},r_{h}\right)\in X_{h}^{k}\times Q_{h}^{k-1}\times Y_{h}^{k}\times W_{0h}^{k}\times S_{h}^{k}\right\}$, ∀$\left\{\left(v,q,\varphi ,C\right)\in X_{h}^{k}\times Q_{h}^{k-1}\times Y_{h}^{k}\times W_{h}^{k}\right\}$ such that

(25) $$\begin{array}{c} A_{1}\left(\nu \left(\theta_{h}\right),u_{h},v\right)+O_{1}\left(u_{h},u_{h},v\right)-\left(\beta \left(\theta_{h}\right)\theta_{h},v\right)\\ +\mu \;O_{2}\left(B_{h},B_{h},v\right)+b\left(v,p_{h}\right)=\left(f,v\right), \end{array}$$

(26) $$\begin{array}{cc} \; & b(u_{h},q)=0, \end{array}$$

(27) $$\begin{array}{cc} \; & A_{2}\left(\sigma \left(\theta_{h}\right),B_{h},C\right)-O_{2}\left(B_{h},C,u_{h}\right)+a\left(C,r_{h}\right)=\left(g,C\right), \end{array}$$

(28) $$\begin{array}{cc} \; & A_{3}\left(\kappa \left(\theta_{h}\right),\theta_{h},\varphi \right)+O_{3}\left(u_{h},\theta_{h},\varphi \right)=\left(\psi ,\varphi \right). \end{array}$$

**Remark** **3.** *The temperature-dependent coefficients will greatly enhance the nonlinearity of the problem and make the analysis more complicated. The existence of a solution to Equations (25)–(28) can be displayed by Brouwer’s fixed point theorem, for details, refer to Section 4.3 of [39].*

**Remark** **4.** *For all $s_{h}\in S_{h}^{k}$, by selecting $C=\nabla s_{h}$ in Equation (27), it can be derived $-\left(\nabla r_{h},\nabla s_{h}\right)=\left(g,\nabla s_{h}\right)$. Thus, for a solenoidal source term g, it is natural to deduce $r_{h}=0$.*

In order to estimate the error in the next section, the stability of the numerical scheme in Equations (25)–(28) should be given here.

**Theorem** **2.** *Let $\left(u_{h},\theta_{h},B_{h}\right)$ be the solution of scheme (25)–(28). Then, it satisfies the following stability:*

(29) $$\left|\;|\;\right|\left(u_{h},B_{h},\theta_{h}\right)\left|\;|\;\right|\leq \frac{{∥l∥}_{*}}{\min\{\nu_{0},\mu \sigma_{0},\kappa_{0}\}}.$$

**Proof.**  The proof is parallel to that of Theorem 1. □

## 4. Convergence Analysis of the Magneto-Heat Coupling Problem

In this section, the convergence of the MHD system coupled the heat equation with variable coefficients is considered by employing the finite element method. We strictly establish optimal error estimates of velocity, pressure, temperature, magnetic induction and Lagrange multiplier under the assumption that the exact solution has low regularity.

Here, it is necessary to make the following regularity assumptions for the weak solution of Equations (7)–(9), which will facilitate the error estimate of the discrete solution.

**Assumption** **1.** *Assum that the solution (u,p,B,θ,r) satisfies the following regularity:*

$$\begin{array}{cc} \; & u\in H^{s+1}\left(\Omega \right),\;p\in H^{s}\left(\Omega \right),\;\theta \in H^{1+s}\left(\Omega \right),\\ \; & B\in H^{s}\left(\Omega \right),\;\mathrm{curl}\;B\in H^{s}\left(\Omega \right),\;r\in H^{s+1}\left(\Omega \right), \end{array}$$

*where the exponent s>1/2 depends on Ω.*

For the convenience of the subsequent analysis, we will assume there exists a constant $C_{f}$ depending on f, g, ψ and Ω such that

(30) $$\begin{array}{c} {∥u∥}_{1+s,2}+{∥p∥}_{s,2}+{∥\mathrm{curl}\;B∥}_{s,2}+{∥\theta ∥}_{1+s,2}+{∥\nabla r∥}_{s,2}\leq C_{f}. \end{array}$$

Let ℓ=min{k,s}, for k≥1 and s>1/2, with the help of [31,40], then we have the following approximation properties:

(31) $$\begin{array}{c} \underset{v\in X_{h}^{k}}{\inf}{∥\nabla \left(u-v\right)∥}_{0}+\underset{q\in Q_{h}^{k-1}}{\inf}{∥p-q∥}_{0}\leq Ch^{\ell }\left[{∥u∥}_{1+\ell ,2}+{∥p∥}_{\ell ,2}\right]\\ \underset{C\in W_{h}^{k}}{\inf}{∥\mathrm{curl}\;\left(B-C\right)∥}_{0}+\underset{j\in S_{h}^{k}}{\inf}{∥\nabla \left(r-j\right)∥}_{0}\leq Ch^{\ell }\left[{∥\mathrm{curl}\;B∥}_{\ell ,2}+{∥r∥}_{1+\ell ,2}\right],\\ \underset{\varphi \in Y_{h}^{k}}{\inf}{∥\nabla \left(\theta -\varphi \right)∥}_{0}\leq Ch^{\ell }{∥\theta ∥}_{\ell +1,2}, \end{array}$$

where *k* and *s* are the order index of the finite element spaces and the regularity of the exact solution, respectively.

Let $\left(e_{u},\;e_{p},\;e_{\theta },\;e_{B},\;e_{r}\right)=\left(u-u_{h},p-p_{h},\theta -\theta_{h},B-B_{h},r-r_{h}\right)$. A combination of Equations (7)–(9) and Equations (25)–(28) yields the following truncation error equations:

(32) $$\begin{array}{c} A_{1}\left(\nu \left(\theta_{h}\right),e_{u},v\right)+A_{1}\left(\nu \left(\theta \right)-\nu \left(\theta_{h}\right),u,v\right)+b\left(v,e_{p}\right)\\ +O_{1}\left(u_{h},e_{u},v\right)+O_{1}\left(e_{u},u,v\right)+\mu \;O_{2}\left(B_{h},e_{B},v\right)+\mu \;O_{2}\left(e_{B},B,v\right)\\ -\left(\beta \left(\theta_{h}\right)e_{\theta },v\right)-\left(\left[\beta \left(\theta \right)-\beta \left(\theta_{h}\right)\right]\theta ,v\right)=0, \end{array}$$

(33) $$A_{3}\left(\kappa \left(\theta_{h}\right),e_{\theta },\varphi \right)+A_{3}\left(\kappa \left(\theta \right)-\kappa \left(\theta_{h}\right),\theta ,\varphi \right)+O_{3}\left(u_{h},e_{\theta },\varphi \right)+O_{3}\left(e_{u},\theta ,\varphi \right)=0,$$

(34) $$\begin{array}{c} A_{2}\left(\sigma \left(\theta_{h}\right),e_{B},C\right)+A_{2}\left(\sigma \left(\theta \right)-\sigma \left(\theta_{h}\right),B,C\right)\\ -O_{2}\left(e_{B},C,u_{h}\right)-O_{2}\left(B,C,e_{u}\right)+a\left(C,e_{r}\right)=0. \end{array}$$

With the preparations of the above work, we now begin to study the optimal error estimation of each variable.

**Theorem** **3.** *Provided that*

(35) $$\frac{\max\{1,\mu ,C_{lip},\mu C_{lip},\beta_{1}\}}{\min\{\nu_{0},\mu \sigma_{0},\kappa_{0}\}}\max\left\{\frac{{∥l∥}_{*}}{\min\{\nu_{0},\mu \sigma_{0},\kappa_{0}\}},C_{f}\right\}<1$$

*is satisfied. Then, the weak formulation made up of Equations (7)–(9) and the discretization scheme in Equations (25)–(28) has a unique solution $\left(u,p,\theta ,B,r\right)\in X\times Q\times Y\times W_{0}\times H_{0}^{1}\left(\Omega \right)$ and $\left(u_{h},p_{h},\theta_{h},B_{h},r_{h}\right)\in X_{h}^{k}\times Q_{h}^{k-1}\times Y_{h}^{k}\times W_{0h}^{k}\times S_{h}^{k}$, respectively, which satisfies*

$$\begin{array}{cc} |\;|\;|(u-u_{h},B-B_{h},\theta -\theta_{h})|\;|\;|\leq & C\underset{\left(v,C,\varphi \right)\in X_{h}^{k}\times W_{h}^{k}\times Y_{h}^{k}}{\inf}\left|\;|\;|\left(v-u,C-B,\varphi -\theta \right)|\;|\;\right|\\ \; & +C\underset{\left(q,j\right)\in Q_{h}^{k-1}\times S_{h}^{k}}{\inf}|\;|\;|\left(p-q,r-j\right)|\;|\;|. \end{array}$$

**Proof.**  As a first step, the test functions of the momentum and magnetic equations are constrained in the discrete kernel space. Let $\left(v,C\right)\in X_{0h}^{k}\times W_{0h}^{k}$. Using the orthogonality property, with Equation (32), we have

(36) $$\begin{array}{cc} \; & A_{1}\left(\nu \left(\theta_{h}\right),v-u_{h},v-u_{h}\right)+A_{1}\left(\nu \left(\varphi \right)-\nu \left(\theta_{h}\right),u,v-u_{h}\right)\\ \; & +O_{1}\left(u_{h},v-u_{h},v-u_{h}\right)+O_{1}\left(v-u_{h},u,v-u_{h}\right)\\ \; & +\mu \left(B_{h}\times \mathrm{curl}\;\left[C-B_{h}\right],v-u_{h}\right)+\mu \left(\left[C-B_{h}\right]\times \mathrm{curl}\;B,v-u_{h}\right)\\ \; & -\left(\beta \left(\theta_{h}\right)\left[\varphi -\theta_{h}\right],v-u_{h}\right)-\left(\left[\beta \left(\varphi \right)-\beta \left(\theta_{h}\right)\right]\theta ,v-u_{h}\right)\\ = & A_{1}\left(\nu \left(\theta_{h}\right),v-u,v-u_{h}\right)+A_{1}\left(\nu \left(\varphi \right)-\nu \left(\theta \right),u,v-u_{h}\right)-b\left(v-u_{h},p-p_{h}\right)\\ \; & +O_{1}\left(u_{h},v-u,v-u_{h}\right)+O_{1}\left(v-u,u,v-u_{h}\right)\\ \; & +\mu \left(B_{h}\times \mathrm{curl}\;\left[C-B\right],v-u_{h}\right)+\mu \left(\left[C-B\right]\times \mathrm{curl}\;B,v-u_{h}\right)\\ \; & -\left(\beta \left(\theta_{h}\right)\left[\varphi -\theta \right],v-u_{h}\right)-\left(\left[\beta \left(\varphi \right)-\beta \left(\theta \right)\right]\theta ,v-u_{h}\right). \end{array}$$

According to Lemma 1 and the property of Hodge mapping (Equation (21)), by setting $1/\left(3+\delta_{1}\right)+1/\left(6-\delta_{2}\right)=1/2$, it is easy to see that

$$\begin{array}{cc} \; & \left|\mu (B_{h}\times \mathrm{curl}\;\left[C-B_{h}\right],v-u_{h})\right|\\ = & \left|\mu (\left[B_{h}-Z_{h}\left(B_{h}\right)\right]\times \mathrm{curl}\;\left[C-B_{h}\right],v-u_{h}\right)\\ \; & +\mu (Z_{h}\left(B_{h}\right)\times \mathrm{curl}\;\left[C-B_{h}\right],v-u_{h})|\\ \leq & \mu ∥B_{h}-Z_{h}\left(B_{h}\right){∥}_{0}∥\mathrm{curl}\;\left[C-B_{h}\right]{∥}_{0}{∥v-u_{h}∥}_{0,\infty }\\ \; & +\mu ∥Z_{h}\left(B_{h}\right){∥}_{0,3+\delta_{1}}∥\mathrm{curl}\;\left[C-B_{h}\right]{∥}_{0}{∥v-u_{h}∥}_{0,6-\delta_{2}}\\ \leq & \mu C_{inv}h^{l}∥\mathrm{curl}\;B_{h}{∥}_{0}∥\mathrm{curl}\;\left[C-B_{h}\right]{∥}_{0}{∥v-u_{h}∥}_{0,6}\\ \; & +\mu ∥\mathrm{curl}\;B_{h}{∥}_{0}∥\mathrm{curl}\;\left[C-B_{h}\right]{∥}_{0}{∥v-u_{h}∥}_{0,6-\delta_{2}}, \end{array}$$

and similarly

$$\begin{array}{cc} \; & \left|\mu (\left[C-B_{h}\right]\times \mathrm{curl}\;B,v-u_{h})\right|\\ = & \left|\mu (\left[C-B_{h}-Z_{h}\left(C-B_{h}\right)\right]\times \mathrm{curl}\;B,v-u_{h}\right)\\ \; & +\mu (Z_{h}\left(C-B_{h}\right)\times \mathrm{curl}\;B,v-u_{h})|\\ \leq & \mu C_{inv}h^{l}∥\mathrm{curl}\;\left[C-B_{h}\right]{∥}_{0}{∥\mathrm{curl}\;B∥}_{0}{∥v-u_{h}∥}_{0,6}\\ \; & +\mu ∥\mathrm{curl}\;\left[C-B_{h}\right]{∥}_{0}{∥\mathrm{curl}\;B∥}_{0}{∥v-u_{h}∥}_{0,6-\delta_{2}}. \end{array}$$

As a consequence of the previous calculation, we can estimate Equation (36) as follows

(37) $$\begin{array}{cc} \; & [\nu_{0}-C_{lip}{∥\nabla u∥}_{0,3}-C_{*}{∥\nabla u∥}_{0}-\mu \left(C_{inv}h^{l}+1\right)\left[∥\mathrm{curl}\;B_{h}{∥}_{0}+{∥\mathrm{curl}\;B∥}_{0}\right]\\ \; & -\beta_{1}-C_{lip}{∥\theta ∥}_{0,3}][∥\nabla \left(v-u_{h}\right){∥}_{0}^{2}+∥\nabla \left(\varphi -\theta_{h}\right){∥}_{0}{∥\nabla \left(v-u_{h}\right)∥}_{0}\\ \; & +∥\mathrm{curl}\;\left[C-B_{h}\right]{∥}_{0}{∥\nabla \left(v-u_{h}\right)∥}_{0}]\\ \leq & A_{1}\left(\nu \left(\theta_{h}\right),v-u_{h},v-u_{h}\right)+A_{1}\left(\nu \left(\varphi \right)-\nu \left(\theta_{h}\right),u,v-u_{h}\right)\\ \; & +O_{1}\left(v-u_{h},u,v-u_{h}\right)\\ \; & +\mu \left(B_{h}\times \mathrm{curl}\;\left[C-B_{h}\right],v-u_{h}\right)+\mu \left(\left[C-B_{h}\right]\times \mathrm{curl}\;B,v-u_{h}\right)\\ \; & -\left(\beta \left(\theta_{h}\right)\left[\varphi -\theta_{h}\right],v-u_{h}\right)-\left(\left[\beta \left(\varphi \right)-\beta \left(\theta_{h}\right)\right]\theta ,v-u_{h}\right). \end{array}$$

Since $v-u_{h}$ belongs to kernel space $X_{0h}^{k}$, we deduce $b\left(v-u_{h},p-p_{h}\right)=b\left(v-u_{h},p-q\right)$ for any $q\in Q_{h}^{k-1}$. The right-hand side of Equation (36) has the following estimate

(38) $$\begin{array}{cc} \; & A_{1}\left(\nu \left(\theta_{h}\right),v-u,v-u_{h}\right)+A_{1}\left(\nu \left(\varphi \right)-\nu \left(\theta \right),u,v-u_{h}\right)-b\left(v-u_{h},p-p_{h}\right)\\ \; & +O_{1}\left(u_{h},v-u,v-u_{h}\right)+O_{1}\left(v-u,u,v-u_{h}\right)\\ \; & +\mu \left(B_{h}\times \mathrm{curl}\;\left[C-B\right],v-u_{h}\right)+\mu \left(\left[C-B\right]\times \mathrm{curl}\;B,v-u_{h}\right)\\ \; & -\left(\beta \left(\theta_{h}\right)\left[\varphi -\theta \right],v-u_{h}\right)-\left(\left[\beta \left(\varphi \right)-\beta \left(\theta \right)\right]\theta ,v-u_{h}\right)\\ \leq & \nu_{1}{∥\nabla \left(v-u\right)∥}_{0}∥\nabla \left(v-u_{h}\right){∥}_{0}+C_{lip}{∥\nabla u∥}_{0,3}{∥\varphi -\theta ∥}_{0,6}{∥\nabla \left(v-u_{h}\right)∥}_{0}\\ \; & +\beta_{*}∥\nabla \left(v-u_{h}\right){∥}_{0}{∥p-q∥}_{0}+C_{*}\left[{∥\nabla u∥}_{0}+{∥\nabla u_{h}∥}_{0}\right]∥\nabla \left(v-u\right){∥}_{0}{∥\nabla \left(v-u_{h}\right)∥}_{0}\\ \; & +\mu \left(C_{inv}h^{l}+1\right)\left[∥\mathrm{curl}\;B_{h}{∥}_{0}+{∥\mathrm{curl}\;B∥}_{0}\right]∥\mathrm{curl}\;\left[C-B\right]{∥}_{0}{∥v-u_{h}∥}_{0,6}\\ \; & +\beta_{1}{∥\varphi -\theta ∥}_{0}∥v-u_{h}{∥}_{0}+C_{lip}{∥\theta ∥}_{0,3}{∥\varphi -\theta ∥}_{0,6}{∥v-u_{h}∥}_{0}. \end{array}$$

Using Equation (33), we can arrive at

(39) $$\begin{array}{cc} \; & A_{3}\left(\kappa \left(\theta_{h}\right),\varphi -\theta_{h},\varphi -\theta_{h}\right)+A_{3}\left(\kappa \left(\varphi \right)-\kappa \left(\theta_{h}\right),\theta ,\varphi -\theta_{h}\right)\\ \; & +O_{3}\left(u_{h},\varphi -\theta_{h},\varphi -\theta_{h}\right)+O_{3}\left(v-u_{h},\theta ,\varphi -\theta_{h}\right)\\ = & A_{3}\left(\kappa \left(\theta_{h}\right),\varphi -\theta ,\varphi -\theta_{h}\right)+A_{3}\left(\kappa \left(\varphi \right)-\kappa \left(\theta \right),\theta ,\varphi -\theta_{h}\right)\\ \; & +O_{3}\left(u_{h},\varphi -\theta ,\varphi -\theta_{h}\right)+O_{3}\left(v-u,\theta ,\varphi -\theta_{h}\right). \end{array}$$

The left-hand side of Equation (39) has the following estimate

(40) $$\begin{array}{cc} \; & \left[\kappa_{0}-C_{lip}{∥\nabla \theta ∥}_{0,3}-C_{*}{∥\nabla \theta ∥}_{0}\right]\left[∥\nabla \left(\varphi -\theta_{h}\right){∥}_{0}^{2}+∥\nabla \left(\varphi -\theta_{h}\right){∥}_{0}{∥\nabla \left(v-u_{h}\right)∥}_{0}\right]\\ \leq & A_{3}\left(\kappa \left(\theta_{h}\right),\varphi -\theta_{h},\varphi -\theta_{h}\right)+A_{3}\left(\kappa \left(\varphi \right)-\kappa \left(\theta_{h}\right),\theta ,\varphi -\theta_{h}\right)\\ \; & +O_{3}\left(v-u_{h},\theta ,\varphi -\theta_{h}\right). \end{array}$$

The right-hand side of Equation (39) has the following estimate

(41) $$\begin{array}{cc} \; & A_{3}\left(\kappa \left(\theta_{h}\right),\varphi -\theta ,\varphi -\theta_{h}\right)+A_{3}\left(\kappa \left(\varphi \right)-\kappa \left(\theta \right),\theta ,\varphi -\theta_{h}\right)\\ \; & +O_{3}\left(u_{h},\varphi -\theta ,\varphi -\theta_{h}\right)+O_{3}\left(v-u,\theta ,\varphi -\theta_{h}\right)\\ \leq & \kappa_{1}{∥\nabla \left(\varphi -\theta \right)∥}_{0}∥\nabla \left(\varphi -\theta_{h}\right){∥}_{0}+C_{lip}{∥\nabla \theta ∥}_{0,3}{∥\varphi -\theta ∥}_{0,6}{∥\nabla \left(\varphi -\theta_{h}\right)∥}_{0}\\ \; & +C_{*}\left[∥\nabla u_{h}{∥}_{0}{∥\nabla \left(\varphi -\theta \right)∥}_{0}+{∥\nabla \theta ∥}_{0}{∥\nabla \left(v-u\right)∥}_{0}\right]{∥\nabla \left(\varphi -\theta_{h}\right)∥}_{0}. \end{array}$$

Using Equation (34), we derive

(42) $$\begin{array}{cc} \; & A_{2}\left(\sigma \left(\theta_{h}\right),C-B_{h},C-B_{h}\right)+A_{2}\left(\sigma \left(\varphi \right)-\sigma \left(\theta_{h}\right),B,C-B_{h}\right)r\\ \; & -\left(u_{h}\times \left[C-B_{h}\right],\mathrm{curl}\;\left[C-B_{h}\right]\right)-\left(\left[v-u_{h}\right]\times B,\mathrm{curl}\;\left[C-B_{h}\right]\right)\\ = & A_{2}\left(\sigma \left(\theta_{h}\right),C-B,C-B_{h}\right)+A_{2}\left(\sigma \left(\varphi \right)-\sigma \left(\theta \right),B,C-B_{h}\right)\\ \; & -\left(u_{h}\times \left[C-B\right],\mathrm{curl}\;\left[C-B_{h}\right]\right)-\left(\left[v-u\right]\times B,\mathrm{curl}\;\left[C-B_{h}\right]\right)\\ \; & +(\nabla e_{r},C-B_{h}). \end{array}$$

The left-hand side of Equation (42) has the following estimate

(43) $$\begin{array}{cc} \; & \left[\sigma_{0}\mu -C_{lip}{\mu ∥\mathrm{curl}\;B∥}_{0,3}-\mu \left(C_{inv}h^{l}+1\right)(∥\nabla u_{h}{∥}_{0}+{∥\mathrm{curl}\;B∥}_{0})\right]\\ \; & [∥\mathrm{curl}\;\left(C-B_{h}\right){∥}_{0}^{2}+∥\nabla \left(\varphi -\theta_{h}\right){∥}_{0}{∥\mathrm{curl}\;\left(C-B_{h}\right)∥}_{0}\\ \; & +∥\nabla \left(v-u_{h}\right){∥}_{0}{∥\mathrm{curl}\;\left(C-B_{h}\right)∥}_{0}]\\ \leq & \mu A_{2}\left(\sigma \left(\theta_{h}\right),C-B_{h},C-B_{h}\right)+\mu A_{2}\left(\sigma \left(\varphi \right)-\sigma \left(\theta_{h}\right),B,C-B_{h}\right)\\ \; & -\mu (u_{h}\times \left[C-B_{h}\right],\mathrm{curl}\;\left[C-B_{h}\right])\\ \; & -\mu (\left[v-u_{h}\right]\times B,\mathrm{curl}\;\left[C-B_{h}\right]). \end{array}$$

Since $C-B_{h}$ belongs to the kernel space $W_{0h}^{k}$, we have $\left(\nabla e_{r},C-B_{h}\right)=\left(\nabla \left(r-j\right),C-B_{h}\right)$ for any $j\in S_{h}^{k}$. The right-hand side of Equation (42) has the following estimate

(44) $$\begin{array}{cc} \; & \mu A_{2}\left(\sigma \left(\theta_{h}\right),C-B,C-B_{h}\right)+\mu A_{2}\left(\sigma \left(\varphi \right)-\sigma \left(\theta \right),B,C-B_{h}\right)\\ \; & -\mu (u_{h}\times \left[C-B\right],\mathrm{curl}\;\left[C-B_{h}\right])\\ \; & -\mu \left(\left[v-u\right]\times B,\mathrm{curl}\;\left[C-B_{h}\right]\right)+\mu \left(\nabla e_{r},C-B_{h}\right)\\ \leq & \sigma_{1}{\mu ∥\mathrm{curl}\;\left(C-B\right)∥}_{0}{∥\mathrm{curl}\;\left(C-B_{h}\right)∥}_{0}\\ \; & +C_{lip}{\mu ∥\mathrm{curl}\;B∥}_{0,3}{∥\varphi -\theta ∥}_{0,6}{∥\mathrm{curl}\;\left(C-B_{h}\right)∥}_{0}\\ \; & +\mu \;[C_{inv}h^{l}∥\nabla u_{h}{∥}_{0}{∥\mathrm{curl}\;\left(C-B\right)∥}_{0}\\ \; & +{∥\mathrm{curl}\;B∥}_{0}{∥\nabla \left(v-u\right)∥}_{0}]{∥\mathrm{curl}\;\left(C-B_{h}\right)∥}_{0}\\ \; & +\mu \beta_{*}∥\mathrm{curl}\;\left(C-B_{h}\right){∥}_{0}{∥\nabla \left(r-j\right)∥}_{0}. \end{array}$$

Combining Equations (37), (40), (43), (38), (41) and (44), together with (32)–(34), it can be checked that

$$\begin{array}{cc} \; & \left[\min\left\{\nu_{0},\mu \sigma_{0},\kappa_{0}\right\}-\max\left\{1,\mu ,C_{lip},\mu C_{lip},\beta_{1}\right\}\max\left\{\frac{{∥l∥}_{*}}{\min\{\nu_{0},\mu \sigma_{0},\kappa_{0}\}},C_{f}\right\}\right]\\ \; & (∥\nabla \left(v-u_{h}\right){∥}_{0}^{2}+∥\nabla \left(\varphi -\theta_{h}\right){∥}_{0}∥\nabla \left(v-u_{h}\right){∥}_{0}+∥\mathrm{curl}\;\left(C-B_{h}\right){∥}_{0}{∥\nabla \left(v-u_{h}\right)∥}_{0}\\ \; & +∥\nabla \left(\varphi -\theta_{h}\right){∥}_{0}^{2}+∥\mathrm{curl}\;\left(C-B_{h}\right){∥}_{0}^{2}+∥\nabla \left(\varphi -\theta_{h}\right){∥}_{0}{∥\mathrm{curl}\;\left(C-B_{h}\right)∥}_{0})\\ \leq & {C[∥\nabla \left(v-u\right)∥}_{0}∥\nabla \left(v-u_{h}\right){∥}_{0}+∥\nabla \left(\varphi -\theta \right){∥}_{0}{∥\nabla \left(v-u_{h}\right)∥}_{0}\\ \; & {+∥p-q∥}_{0}∥\nabla \left(v-u_{h}\right){∥}_{0}+∥\mathrm{curl}\;\left(C-B\right){∥}_{0}{∥\nabla \left(v-u_{h}\right)∥}_{0}\\ \; & +{∥\nabla \left(\varphi -\theta \right)∥}_{0}∥\nabla \left(\varphi -\theta_{h}\right){∥}_{0}+∥\nabla \left(v-u\right){∥}_{0}{∥\nabla \left(\varphi -\theta_{h}\right)∥}_{0}\\ \; & +{∥\mathrm{curl}\;\left(C-B\right)∥}_{0}∥\mathrm{curl}\;\left(C-B_{h}\right){∥}_{0}+∥\nabla \left(\varphi -\theta \right){∥}_{0}{∥\mathrm{curl}\;\left(C-B_{h}\right)∥}_{0}\\ \; & +{∥\nabla \left(v-u\right)∥}_{0}∥\mathrm{curl}\;\left(C-B_{h}\right){∥}_{0}+∥\mathrm{curl}\;\left(C-B_{h}\right){∥}_{0}{∥\nabla \left(r-j\right)∥}_{0}]. \end{array}$$

Since

$$\begin{array}{c} \min\left\{\nu_{0},\mu \sigma_{0},\kappa_{0}\right\}-\max\left\{1,\mu ,C_{lip},\mu C_{lip},\beta_{1}\right\}\max\left\{\frac{{∥l∥}_{*}}{\min\{\nu_{0},\mu \sigma_{0},\kappa_{0}\}},C_{f}\right\}>0, \end{array}$$

this means that

$$\begin{array}{cc} \; & \left|\;|\;\right|\left(v-u_{h},C-B_{h},\varphi -\theta_{h}\right){\left|\;|\;\right|}^{2}\\ \leq & C|\;|\;|\left(v-u_{h},C-B_{h},\varphi -\theta_{h}\right)\left|\;|\;|[|\;|\;\right|\left(v-u,C-B,\varphi -\theta \right)\left|\;|\;\right|\\ \; & {+∥p-q∥}_{0}+{∥\nabla \left(r-j\right)∥}_{0}]. \end{array}$$

By the triangle inequality, there holds

(45) $$\begin{array}{cc} |\;|\;|(u-u_{h},B-B_{h},\theta -\theta_{h})|\;|\;|\leq & C[|\;|\;|(v-u,C-B,\varphi -\theta )|\;|\;|\\ \; & {+∥p-q∥}_{0}+{∥\nabla \left(r-j\right)∥}_{0}], \end{array}$$

for all (v,C) belongs to the discrete kernel space $\left(X_{0h}^{k},W_{0h}^{k}\right)$, $\varphi \in Y_{h}^{k}$, $q\in Q_{h}^{k-1}$ and $j\in S_{h}^{k}$.

In the next step, let $\left(v,C\right)\in X_{h}^{k}\times W_{h}^{k}$ be arbitrary. Suppose that $\left(w,d\right)\in X_{h}^{k}\times W_{h}^{k}$ is a solution of

$$\begin{array}{c} b(q,w)+a(d,j)=b(q,u-v)+a(B-C,j), \end{array}$$

for any $\left(q,j\right)\in Q_{h}^{k-1}\times S_{h}^{k}$.

From the inf-sup condition and the continuity of b(·,·) and a(·,·), there exists a solution to this problem that satisfies

(46) |||(w,d)|||≤C|||(u−v,B−C)|||.

See [36] for more details. Then, $\left(w+v,d+B\right)\in X_{0h}^{k}\times W_{0h}^{k}$ can be inserted into Equation (45). With the triangle inequality, we deduce

(47) $$\begin{array}{cc} |\;|\;|(u-u_{h},B-B_{h},\theta -\theta_{h})|\;|\;|\leq & C[|\;|\;|(v-u,C-B,\varphi -\theta )|\;|\;|\\ \; & {+|\;|\;|\left(w,d\right)|\;|\;|+∥p-q∥}_{0}+{∥\nabla \left(r-j\right)∥}_{0}]. \end{array}$$

Applying Equations (46) and (47) shows that the proof is completed. □

In addition, by using the inf-sup condition (Equation (23)), we obtain the following estimates for pressure and the Lagrange multiplier.

**Theorem** **4.** *Suppose that Assumption 1 and Theorem 3 are satisfied. The continuous system of Equations (7)–(9) and the numerical scheme in Equations (25)–(28) has the unique solution (p,r) and $\left(p_{h},r_{h}\right)$, respectively, which satisfies*

$$\begin{array}{cc} ∥p-p_{h}{∥}_{0}+{∥\nabla \left(r-r_{h}\right)∥}_{0}\leq & C\underset{\left(v,C,\varphi \right)\in X_{h}^{k}\times W_{h}^{k}\times Y_{h}^{k}}{\inf}\left|\;|\;|\left(v-u,C-B,\varphi -\theta \right)|\;|\;\right|\\ \; & +C\underset{q\in Q_{h}^{k-1}}{\inf}{∥p-q∥}_{0}+C\underset{j\in S_{h}^{k}}{\inf}{∥\nabla \left(r-j\right)∥}_{0}. \end{array}$$

**Proof.**  From Equations (32) and (34), we know

$$\begin{array}{cc} ∥p-p_{h}{∥}_{0}+{∥\nabla \left(r-r_{h}\right)∥}_{0}\leq & C|\;|\;|\left(u-u_{h},B-B_{h},\theta -\theta_{h}\right)|\;|\;|(\nu_{1}+C_{lip}{∥\nabla u∥}_{0,3}\\ \; & +C_{lip}{∥\mathrm{curl}\;B∥}_{0,3}+\left|\;|\;|\left(u,B,\theta \right)|\;|\;\right|+\left|\;|\;\right|\left(u_{h},B_{h}\right)\left|\;|\;\right|+\beta_{1}). \end{array}$$

Combining Assumption 1 and Theorem 3, we can conclude Theorem 4. □

With the help of (31), the following theorem draws the conclusion of this paper.

**Theorem** **5.** *Suppose that Assumption 1, Theorem 3 and Theorem 4 are satisfied. With ℓ=min{s,k}, we have*

$$\begin{array}{cc} \; & ∥\nabla \left(u-u_{h}\right){∥}_{0}+∥p-p_{h}{∥}_{0}+{∥\mathrm{curl}\;\left(B-B_{h}\right)∥}_{0}\\ \; & +∥\nabla \left(r-r_{h}\right){∥}_{0}+{∥\nabla \left(\theta -\theta_{h}\right)∥}_{0}\\ \leq & Ch^{\ell }\left({∥u∥}_{\ell +1,2}+{∥p∥}_{\ell ,2}+{∥\mathrm{curl}\;B∥}_{\ell ,2}+{∥\theta ∥}_{\ell +1,2}+{∥\nabla r∥}_{\ell ,2}\right). \end{array}$$

## 5. Numerical Experiment

In this section, we consider a numerical experiment to test the convergence rate of the numerical scheme proposed in Section 3. The parallel code is developed based on the finite element package Parallel Hierarchical Grids (PHG), cf. [41,42]. The computations are carried out on the LSSC-IV Cluster of the State Key Laboratory of Scientific and Engineering Computing, Chinese Academy of Sciences. The domain is $\Omega ={\left(0,1\right)}^{3}$, and the finite element mesh is obtained by a uniform tetrahedral partition. Let $T_{i},i=0,1,2,3$ be four successively refined meshes listed in Table 1.

**Example** **1.** *This example is to verify the convergence rate of the finite element solution. Given ν(θ)=1,σ(θ)=θ,κ(θ)=θ, μ=1 and β(θ)=(0,0,−1). The exact solution is selected as*

$$\begin{array}{cc} \; & u=(\sin(y),\sin(z),0),\;p=\sin(x)-\sin(y),\\ \; & B=(\cos(y),\cos(z),0),\;r=0,\;\theta =\sin(x). \end{array}$$

*From Table 2 and Table 3, we find that the convergence rates for $u_{h},p_{h},B_{h}$ and $\theta_{h}$ are given by*

$$\begin{array}{cc} \; & ∥u-u_{h}{∥}_{1,2}∼O\left(h^{2}\right),\;∥u-u_{h}{∥}_{0}∼O\left(h^{3}\right),\;{∥p-p_{h}∥}_{0}∼O\left(h^{2}\right),\\ \; & ∥B-B_{h}{∥}_{H(\mathrm{curl};\Omega )}∼O\left(h\right),\;{∥B-B_{h}∥}_{0}∼O\left(h^{2}\right),\\ \; & ∥\theta -\theta_{h}{∥}_{1,2}∼O\left(h^{2}\right),\;{∥\theta -\theta_{h}∥}_{0}∼O\left(h^{3}\right). \end{array}$$

*Here, the velocity u and the temperature θ are discretized by using the continuous $P_{2}$ finite elements, the pressure p is discretized by using the continuous $P_{1}$ finite elements and the magnetic induction B is discretized by using the first-order edge elements method. This means that optimal convergence rates are obtained for all variables.*

## Figures and Tables

**Table 1:** Numbers of DOFs on four successively refined meshes.

Grid	*h*	DOFs for $\left(u_{h},p_{h}\right)$	DOFs for $\left(B_{h},r_{h}\right)$	DOFs for $\left(\theta_{h}\right)$	Ndofs
$T_{0}$	0.866	402	321	125	848
$T_{1}$	0.433	2312	1937	729	4978
$T_{2}$	0.217	15,468	13,281	4913	33,662
$T_{3}$	0.108	112,724	97,985	35,937	246,646

**Table 2:** Convergence rates in energy norms.

*h*	$∥u-u_{h}{∥}_{1,2}$	order	$∥p-p_{h}{∥}_{0}$	order
0.866	1.136 $\times \;10^{-2}$	- -	1.187 $\times \;10^{-2}$	- -
0.433	2.745 $\times \;10^{-3}$	2.0488	2.496 $\times \;10^{-3}$	2.2501
0.217	6.766 $\times \;10^{-4}$	2.0271	5.774 $\times \;10^{-4}$	2.1189
0.108	1.686 $\times \;10^{-4}$	1.9919	1.377 $\times \;10^{-4}$	2.0545
*h*	$∥B-B_{h}{∥}_{H(\mathrm{curl};\Omega )}$	order	$∥\theta -\theta_{h}{∥}_{1,2}$	order
0.866	1.405 $\times \;10^{-1}$	- -	7.545 $\times \;10^{-3}$	- -
0.433	6.873 $\times \;10^{-2}$	1.0315	1.837 $\times \;10^{-3}$	2.0384
0.217	3.382 $\times \;10^{-2}$	1.0263	4.456 $\times \;10^{-4}$	2.0500
0.108	1.677 $\times \;10^{-2}$	1.0057	1.089 $\times \;10^{-4}$	2.0194

**Table 3:** Convergence rates in $L^{2}\left(\Omega \right)$—norms.

*h*	$∥u-u_{h}{∥}_{0}$	Order	$∥\theta -\theta_{h}{∥}_{0}$	Order	$∥B-B_{h}{∥}_{0}$	Order
0.866	9.095 $\times \;10^{-4}$	- -	5.995 $\times \;10^{-4}$	- -	1.851 $\times \;10^{-2}$	- -
0.433	1.136 $\times \;10^{-4}$	3.0010	7.494 $\times \;10^{-5}$	2.9999	4.977 $\times \;10^{-3}$	1.8950
0.217	1.442 $\times \;10^{-5}$	2.9883	9.376 $\times \;10^{-6}$	3.0088	1.268 $\times \;10^{-3}$	1.9794
0.108	1.950 $\times \;10^{-6}$	2.8671	1.142 $\times \;10^{-6}$	3.0169	3.178 $\times \;10^{-4}$	1.9830

## Data Availability

Not applicable.
